# VlsE, the nexus for antigenic variation of the Lyme disease spirochete, also mediates early bacterial attachment to the host microvasculature under shear force

**DOI:** 10.1371/journal.ppat.1010511

**Published:** 2022-05-23

**Authors:** Xi Tan, Yi-Pin Lin, Michael J. Pereira, Mildred Castellanos, Beth L. Hahn, Phillip Anderson, Jenifer Coburn, John M. Leong, George Chaconas

**Affiliations:** 1 Department of Biochemistry & Molecular Biology, Snyder Institute for Chronic Diseases, University of Calgary, Calgary, Alberta, Canada; 2 Division of Infectious Diseases, New York State Department of Health, Wadsworth Center, Albany, New York, United States of America; 3 Department of Molecular Biology and Microbiology, Tufts University School of Medicine, Boston, Massachusetts, United States of America; 4 Department of Medicine, Division of Infectious Diseases, Medical College of Wisconsin, Milwaukee, Wisconsin, United States of America; 5 Departments of Biochemistry & Molecular Biology and Microbiology, Immunology & Infectious Diseases, Snyder Institute for Chronic Diseases, University of Calgary, Calgary, Alberta, Canada; Texas A&M University College Station: Texas A&M University, UNITED STATES

## Abstract

Hematogenous dissemination is a critical step in the evolution of local infection to systemic disease. The Lyme disease (LD) spirochete, which efficiently disseminates to multiple tissues, has provided a model for this process, in particular for the key early event of pathogen adhesion to the host vasculature. This occurs under shear force mediated by interactions between bacterial adhesins and mammalian cell-surface proteins or extracellular matrix (ECM). Using real-time intravital imaging of the Lyme spirochete in living mice, we previously identified BBK32 as the first LD spirochetal adhesin demonstrated to mediate early vascular adhesion in a living mouse; however, deletion of *bbk32* resulted in loss of only about half of the early interactions, suggesting the existence of at least one other adhesin (adhesin-X) that promotes early vascular interactions. VlsE, a surface lipoprotein, was identified long ago by its capacity to undergo rapid antigenic variation, is upregulated in the mammalian host and required for persistent infection in immunocompetent mice. In immunodeficient mice, VlsE shares functional overlap with OspC, a multi-functional protein that displays dermatan sulfate-binding activity and is required for joint invasion and colonization. In this research, using biochemical and genetic approaches as well as intravital imaging, we have identified VlsE as adhesin-X; it is a dermatan sulfate (DS) adhesin that efficiently promotes transient adhesion to the microvasculature under shear force via its DS binding pocket. Intravenous inoculation of mice with a low-passage infectious *B*.* burgdorferi* strain lacking both *bbk32* and *vlsE* almost completely eliminated transient microvascular interactions. Comparative analysis of binding parameters of VlsE, BBK32 and OspC provides a possible explanation why these three DS adhesins display different functionality in terms of their ability to promote early microvascular interactions.

## Author summary

Lyme disease is the most common tick-transmitted disease in the northern hemisphere and is caused by the spirochete *Borrelia burgdorferi* and related *Borrelia* species. The constellation of symptoms attributable to this malady results from *B*. *burgdorferi* dissemination throughout the body via the circulatory system, to invade a variety of tissue types. An early step in this process is the slowing of spirochete velocity in the swirling blood flow via specific interactions between adhesive proteins (adhesins) on the spirochete and their binding targets on the lining of the microvasculature. We have studied this process using biochemical and genetic approaches as well by direct observation of spirochetes in the microvasculature of living mice using intravital microscopy. Our findings solve the mystery of the identity of a previously described adhesin-X, which efficiently promotes early microvascular interactions of the Lyme spirochete. We identify the antigenically variable VlsE protein, required for persistent infection, as a dermatan sulfate adhesin that promotes early microvascular interactions in the mouse under shear force.

## Introduction

Lyme disease (LD), caused by multiple genospecies of the spirochete *Borrelia burgdorferi* sensu lato (hereafter *B*. *burgdorferi*), is the most common tick-borne disease in the North America and Europe [1–3]. Following a tick bite, early localized infection is often recognizable by the presence of a painless bulls-eye rash called erythema migrans. Subsequently, in untreated LD patients the spirochetes hematogenously disseminate [4] to several distal organs, including the joints, heart, or the brain and can produce a wide range of symptoms such as Lyme arthritis, carditis and neuroborreliosis.

Hematogenous dissemination is important for pathogenesis by *B*. *burgdorferi*. A key event in this process is pathogen adhesion to the vascular endothelium under shear force and transmigration from the microvasculature into extravascular tissues; this is mediated in part by interactions between bacterial cell-surface adhesion proteins (adhesins) and mammalian cell-surface proteins or extracellular matrix (ECM) [1,5–9]. Like leucocyte migration across the endothelium, *B*. *burgdorferi* vascular adhesion and extravasation are multistage processes initiated by a series of transient microvascular interactions [10–12], followed by endothelial activation and potentiation [13], which lead to vascular transmigration to colonize perivascular tissue [8,14].

An important strategy allowing pathogenesis and persistence by *B*. *burgdorferi* is the ability of the spirochete to escape destruction by the host innate and acquired immune responses; several mechanisms for doing this exist [15]. A key defense mechanism for avoidance of acquired immunity is antigenic variation of VlsE (see [16,17] for recent reviews). VlsE is a well-known variable outer surface lipoprotein that has been shown to be upregulated during mammalian infection [18]. VlsE is required for spirochete survival and persistence in the presence of a host adaptive immune response [19,20]. Immune evasion occurs through continual changes in surface epitopes of VlsE during mammalian infection, driven by nonreciprocal recombination between the *vlsE* gene and 15 silent *vls* cassettes located on the lp28-1 plasmid [16, 17, 21]. VlsE may act as a shield to obscure the epitopes of other surface antigens from host antibodies for immune evasion [22].

Besides the prominent role for VlsE in escaping immune surveillance, a few studies have hinted at other possible functions for this protein. Enrichment of clones from a phage display library of *B*. *burgdorferi* DNA for localization in specific tissues upon intravenous inoculation into mice identified VlsE as a potential borrelial adhesin with vascular adhesive properties [23]. OspC, P66, BmpD and four members of the OspF family were identified as candidate in the same phage display screen and have since been confirmed as functional adhesins (see [1]). Another important paper suggests that OspC and VlsE may share an overlapping essential function. A *B*. *burgdorferi* strain lacking both OspC and VlsE was unable to promote persistent infection of SCID mice, where antigenic variation is not necessary. However, complementation of *ospC* by its provision in the chromosome or on a shuttle vector was able to restore long-term persistence, suggesting that OspC can substitute for VlsE when antigenic variation is not required [24]. More recently, we have shown that OspC is a dermatan sulfate- (DS-) and fibronectin- (FN-) binding adhesin that is required for vascular transmigration and joint colonization in mice [14].

In the current study, we show that similar to OspC, VlsE is an adhesin that binds to DS *in vitro*, and with a similar binding affinity. Using intravital imaging, we present data that VlsE mediates efficient transient microvascular interactions through its DS binding region. Our experiments also demonstrate that a *B*. *burgdorferi* strain lacking both the strong adhesin BBK32 [11,12,25–27] and VlsE displays only background levels of microvascular interactions, thereby revealing two *B*. *burgdorferi* adhesins that promote the large majority of microvascular transient interactions in living mice.

## Results

### Recombinant VlsE binds to DS glycosaminoglycans

**Fig 1:**
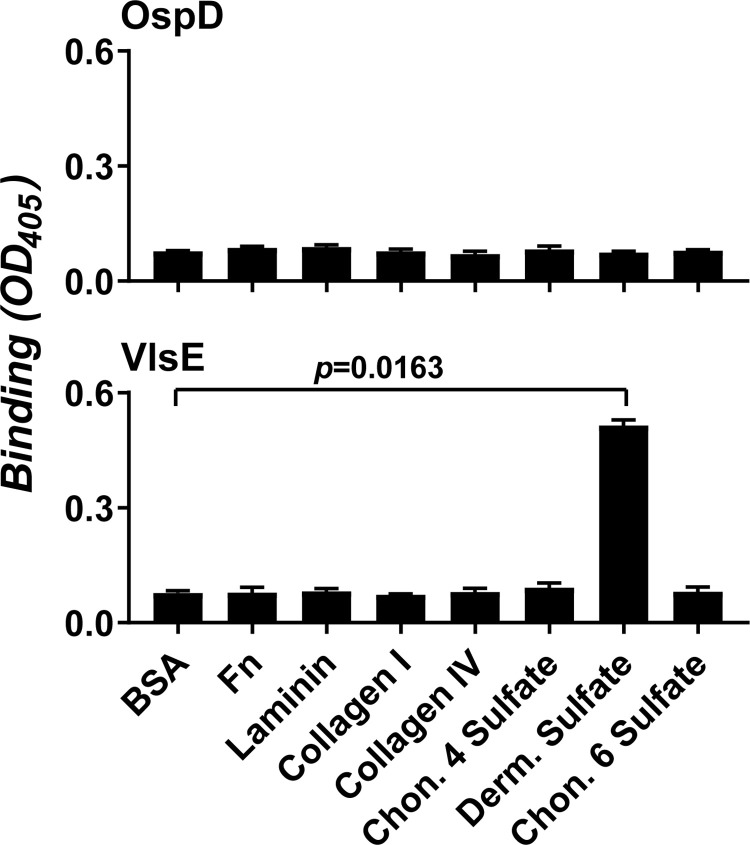
ELISA assay to investigate the binding of VlsE to ECM components. Two μM of recombinant, untagged OspD (top panel, control) or VlsE (bottom panel) from *B*. *burgdorferi* B31 A3 in 100μl of PBS were added in microtiter wells coated with one μg of purified BSA (negative control), fibronectin (FN), laminin, type I collagen (Collagen I), type IV collagen (Collagen IV), chondroitin-4-sulfate (Chon. 4 Sulfate), DS, or chondroitin-6-sulfate (Chon. 6 Sulfate). Protein binding was measured by ELISA in three independent experiments. Each bar represents the mean ± SD of levels of binding from three experiments. Significant differences (P < 0.05, Kruskal-Wallis test with the two-stage step-up method of Benjamini, Krieger, and Yekutieli) in the levels of indicated ligands relative to those of BSA are indicated.

**Fig 2:**
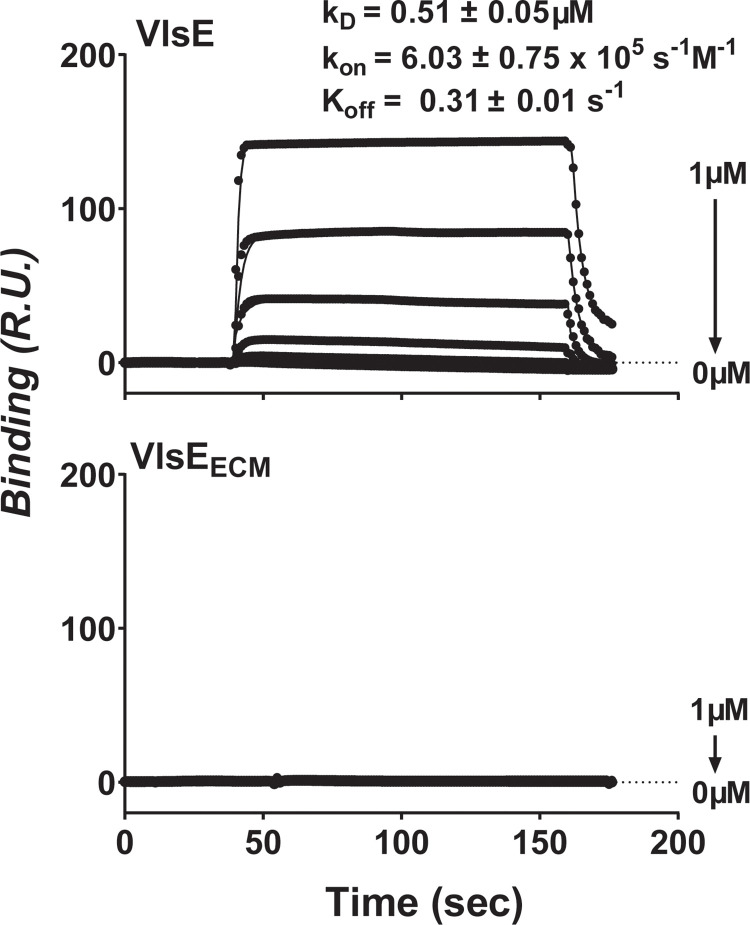
Surface Plasmon Resonance (SPR) to investigate the binding of VlsE to DS. Different concentrations (1000, 250, 62.5, 15.6, 3.9, 0.97, or 0 nM) of recombinant untagged VlsE or a mutated version of VlsE (VlsE_ECM_) were individually flowed over a surface of the SPR chip coated with 10 μg of DS. Binding was measured by SPR. Shown is a representative of six experiments performed on three separate occasions. The mean ± SD of k_on_, k_off_, and apparent K_D_ values for VlsE in binding to DS was obtained from these six experiments and shown on the top of the figure.

To investigate the adhesive properties of VlsE, a recombinant, untagged version of VlsE from *B*. *burgdorferi* B31 strain A3 [28] or a control protein OspD, was produced and added to microtiter wells conjugated with different extracellular matrix molecules or BSA (the negative control protein). These ECMs included FN, laminin, type I collagen, type IV collagen, and glycosaminoglycans (chondroitin-4-sulfate, DS, and chondroitin-6-sulfate). The levels of ligand binding were determined by measuring the absorption values at 405nm (OD_405_) through qualitative ELISA. As expected, neither OspD nor VlsE bound to BSA at levels greater than 0.1 OD_405_, and the binding levels of OspD to any ECM components were not significantly different from those to BSA (**Fig 1 top panel**). However, we found that the binding of VlsE to DS but not any other ECM component was at levels significantly greater than that to BSA (**Fig 1 bottom panel**). We further verified this GAG-binding activity of VlsE under flow conditions using Surface Plasmon Resonance (SPR). By flowing different concentrations of recombinant untagged VlsE through a DS-conjugated chip, we observed a dose-dependent increase in the levels of DS-binding activity with an affinity (apparent K_D_) of 0.51 μM (**Fig 2 top panel**). These results indicate that VlsE is a DS-binding protein.

### Identification of the DS binding region in VlsE

**Fig 3:**
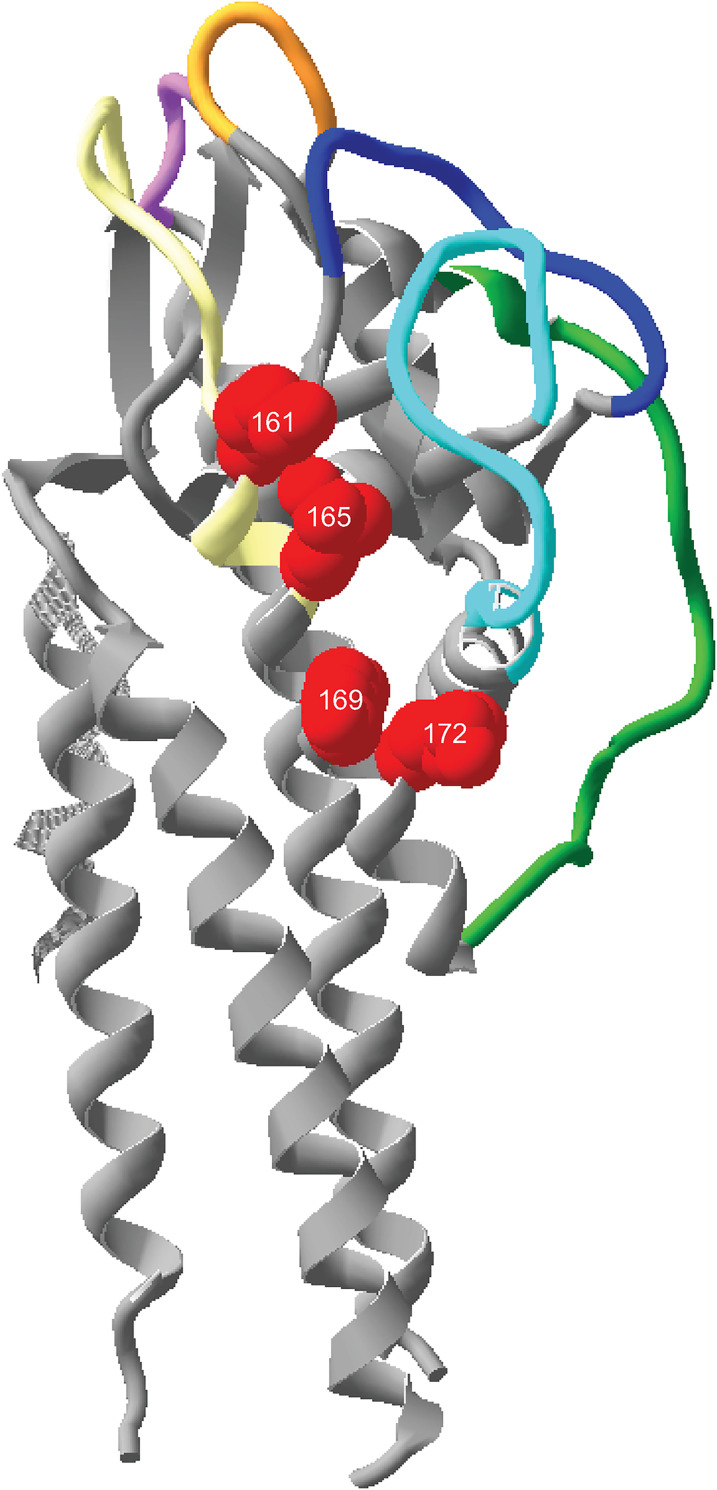
3D structure of VlsE. The three-dimensional structure of VlsE from *B*. *burgdorferi* B31 5A3 (PDB 1L8W) as determined by X-ray crystallography [29] is shown. Yellow, orange, green, magenta, dark blue and light blue depict the surface exposed hypervariable regions where sequence changes occur during antigenic variation [21,31,61]. The membrane associated N-terminal region is at the bottom of the structure. The four lysine residues on the α4 helix predicted to participate in DS binding are shown as red space filling side chains at positions 161, 165, 169 and 172. The VlsE_ECM_ mutant contains methionine residues at these four positions (**Table 1**). Image generated with the Swiss-PDB Viewer.

VlsE was first identified as a potential adhesin by means of a phage display library that was enriched for murine vascular interactions [23]. That report identified a peptide corresponding to a portion of the fourth helix (α4 helix) in the VlsE protein. The entire α4 helix of B31 5A3 VlsE is comprised of 16 amino acids, four of which are lysine residues located on the same face of the helix (**Fig 3,** K-161, K-165, K-169 and K-172) [29]. GAG binding by proteins typically involves clusters of positively charged residues [30] and our previous work with OspC revealed that a cluster of lysine residues is critical for the interaction of OspC with the negatively charged DS [14]. Therefore, we hypothesized that DS binding might be mediated through the lysine residues on the VlsE α4 helix. A VlsE mutant carrying methionine substitutions at positions 161, 165, 169 and 172 was generated (*E*. *coli* strain MP04, see **Table 1)**, removing the positively charged residues while roughly maintaining the size of the amino acid sidechains. This mutant carrying multiple substitutions is referred to as VlsE_ECM_. The four lysines on the α4 helix are not variable during antigenic variation, with the exception of lysine 165, which displays limited variability as only a lysine or threonine [31].

**Table 1:** Bacterial strains used.

Strain number	Background/ Reference	*bbk32*	*vlsE*	Description	Borrelia plasmid Content*	Antibiotic resistance	Source/ Reference
***B*. *burgdorferi* parental strains**							
GCB909 Non-infectious	B31-A [32]				N/A		[32]
GCB919 Non-infectious	B31-5A17 [33]				lp25^-^, lp28^-^		[33]
GCB3022 Infectious	B31-A3 [14]				cp9^-^		[14]
***B*. *burgdorferi* constructed strains**							
GCB705	B31-A [32]			B31-A + pTM61 *gent*, *gfp*		Gm^R^ 100 μg/ml	[12]
GCB726	5A4 NP1 [34]			5A4 NP1 (*kan*)*+* pTM61 *gent*, *gfp*		Km^R^ 200 μg/ml Gm^R^ 100 μg/ml	[10]
GCB3023	B31-A [32]			B31-A + pTM61*strep*,*gfp*,*vlsE*_*A3*_ (prMP09) into GCB909	N/A	Sm^R^ 50 μg/ml	This work
GCB3025	B31-A [32]			B31-A + pTM61*strep*,*gfp*,*vlsE*_*ECM*_ (prMP10) into GCB909	N/A	Sm^R^ 50 μg/ml	This work
GCB3212	B31-A [32]			B31-A + pTM61*strep*,*gfp*		Sm^R^ 50 μg/ml	[8]
GCB4036	B31-5A17 [33]	**-**	**-**	lp25^-^, lp28^-^, Δ*bbk32*::*strep* + pTM61*kan*,*gfp*,*pncA* (pMC119-1, GCE3828) into GCB4465	lp25^-^, lp28^-^, cp9^-^	Km^R^ 200 μg/ml Sm^R^ 50 μg/ml	This work
GCB4043	B31-5A17 [33]	**+**	**-**	lp25^-^, lp28^-^, + pTM61*kan*,*gfp*,*pncA* (pMC119-1, GCE3828) into GCB919	lp25^-^, lp28^-^, cp9^-^	Km^R^ 200 μg/ml	This work
GCB4080	B31-5A17 [33]	**+**	**+**	lp25^-^, lp28^-^, + pTM61*kan*,*gfp*,*pncA*,*vlsE*_*A3*_ (pMC156, GCE4004) into GCB919	lp25^-^, lp28^-^, cp9	Km^R^ 200 μg/ml	This work
GCB4082	B31 5A17 [33]	**-**	**ECM**	lp25^-^, lp28^-^, Δ*bbk32*::*strep* + pTM61*kan*,*gfp*,*pncA*,*vlsE*_*A3-ECM*_ (pTX32, GCE3993) into GCB4465	lp25^-^, lp28^-^, cp9	Km^R^ 200 μg/ml Sm^R^ 50 μg/ml	This work
GCB4452	B31-A3			B31-A3 Δ*ospC* + pTM61*gent*,*gfp*, OspC_B31-ECM_^-^ (pMC115, GCE3817) into GCB3022	cp9^-^	Gm^R^ 100 μg/ml	[14]
GCB4458	B31-A3			B31-A3 Δ*ospC* + pTM61*gent*,*gfp*, OspC_B31_ (pMC114, GCE3815) into GCB3022	lp28-4^-^, lp56^-^, cp9^-^	Gm^R^ 100 μg/ml	[14]
GCB4465	B31-5A17 [33]	**-**	**-**	lp25^-^, lp28^-^, + B31 5A17 *Δbbk32*::*strep* (pMC117, GCE3823) into GCB919	lp25^-^, lp28^-^, lp38^-^, cp9^-^	Sm^R^ 50 μg/ml	This work
GCB4517	B31-5A17 [33]	**-**	**+**	lp25^-^, lp28^-^, Δ*bbk32*::*strep* + pTM61*kan*,*gfp*,*pncA*,*vlsE*_*A3*_ (pMC156, GCE4004) into GCB4465	lp25^-^, lp28^-^, cp9^-^	Km^R^ 200 μg/ml Sm^R^ 50 μg/ml	This work
***E*. *coli* strains**							
MP01	DH5α [35]			DH5α + pGEX4T2, *amp*, *ospD* from *B*. *burgdorferi* strain B31-A3		Amp^R^ 100 μg/ml	This work
MP02	DH5α [35]			DH5α + pGEX4T2, *amp*, *ospD* from *B*. *burgdorferi* strain B31-A3		Amp^R^ 100 μg/ml	This work
MP03	BL21(DE3) [36]			BL21(DE3) + pGEX4T2, *amp*, *vlsE* from *B*. *burgdorferi* strain B31-A3		Amp^R^ 100 μg/ml	This work
MP04	BL21(DE3) [36]			BL21(DE3) + pGEX4T2, *amp*, *vlsE*_*ECM*_ (the VlsE from *B*. *burgdorferi* strain B31-A3 with Lysine-161, -165, -169, and -172 replaced by methionine residues).		Amp^R^ 100 μg/ml	This work
YLT153	BL21(DE3) [36]			BL21(DE3) + pGEX4T2, amp, *ospD* from *B*. *burgdorferi* strain B31-A3		Amp^R^ 100 μg/ml	[37]
GCE3823	DH5α [35]			pMC117 (*Δbbk32*::*strep*) into DH5α		Amp^R^ 100 μg/ml Sm^R^ 25 μg/ml	This work
GCE3828	DH5α [35]			pTM61*kan*,*gfp*,*pncA* (pMC119-1) into DH5α		Km^R^ 50 μg/ml	This work
GCE3951	DH5α [35]			pTM61*kan*,*gfp* (pTM61*kan*-1) into DH5α		Km^R^ 50 μg/ml	This work
GCE3970	DH5α [35]			*pncA +* Topo (pTX19) into DH5α		Km^R^ 50 μg/ml	This work
GCE3993	DH5α [35]			pTM61*kan*,*gfp*,*pncA*,*vlsE*_*A3-ECM*_ (pTX32) into DH5α		Km^R^ 50 μg/ml	This work
GCE3994	DH5α [35]			*vlsE*_*A3-ECM*_ + pJET1.2 (pTX33) into DH5α		Km^R^ 50 μg/ml	This work
GCE4001	DH5α [35]			*vlsE*_*A3*_ + pJET1.2 (pMC154) into DH5α		Amp^R^ 100 μg/ml	This work
GCE4004	DH5α [35]			pTM61*kan*,*gfp*,*pncA*,*vlsE*_*A3*_ (pMC156) into DH5α		Km^R^ 50 μg/ml	This work

To assess possible structural alterations resulting from the methionine substitutions, recombinant untagged VlsE and VlsE_ECM_ were individually analyzed by circular dichroism (CD) spectroscopy. The proportions of the predicted secondary structures of both proteins were calculated according to the spectra with 60.7 and 62.2% of VlsE and VlsE_ECM_, respectively composed of α-helix (**S1 Fig**), in agreement with the helix-rich structure of VlsE [29]. The lack of a significant difference in the secondary structure of the two proteins suggests that the amino acid changes introduced to create VlsE_ECM_ did not change the overall structure of the protein (**S1 Fig**). We also verified that VlsE_ECM_ was localized on the surface of *B*. *burgdorferi*, as was the wild type protein, using a proteinase-K susceptibility assay when expressed *in vivo* (**S2 Fig**).

Finally, we flowed untagged VlsE_ECM_ through the DS-conjugated SPR chip in an increasing concentration (**Fig 2, bottom panel**). No signals of binding were detected, strongly suggesting that the α4 helix of the VlsE lipoprotein forms a positively charged DS binding pocket that binds directly to the negatively charged DS carbohydrate.

### VlsE is an adhesin that promotes transient interaction of *B*. *burgdorferi* with the microvasculature in a high-passage gain-of-function strain

**Fig 4:**
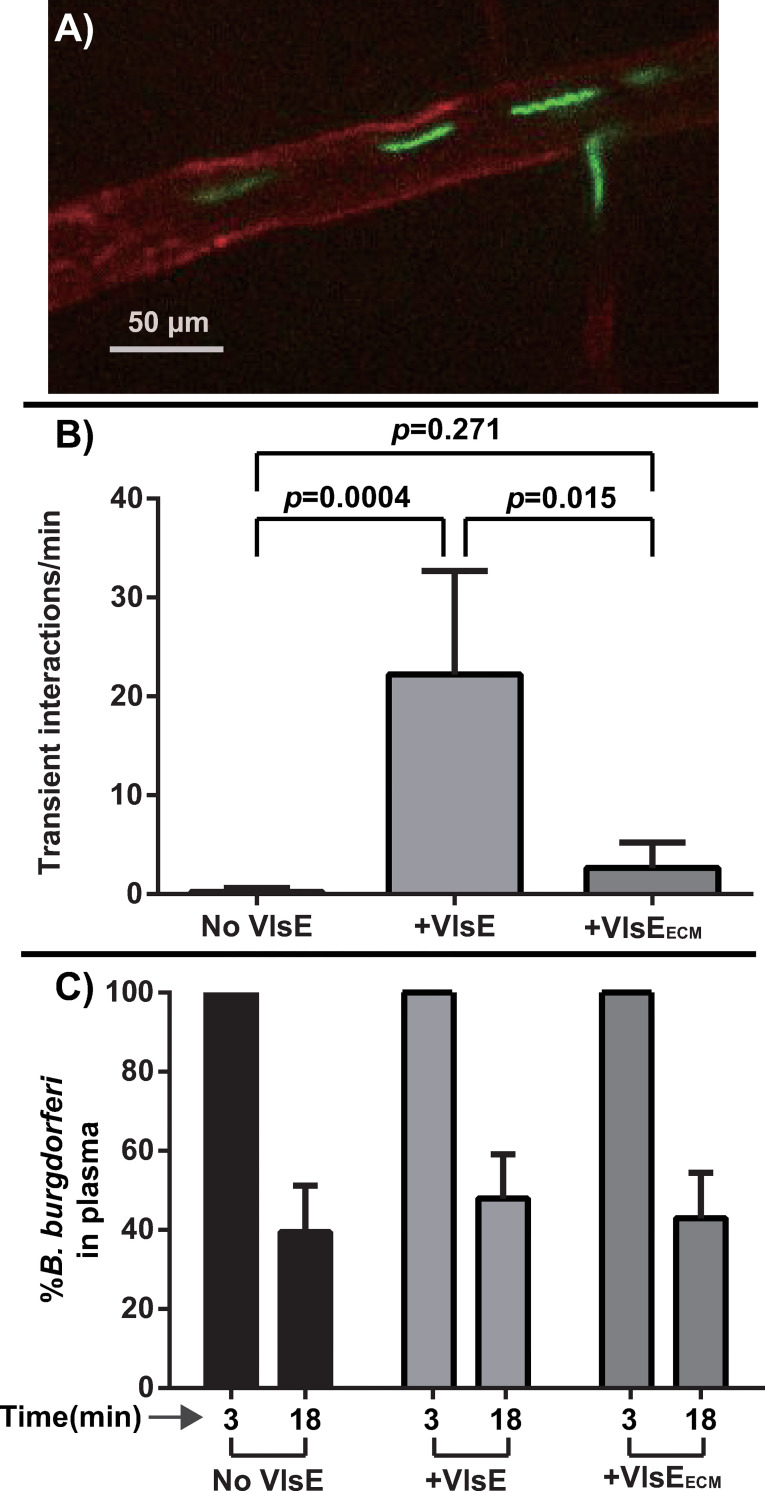
Intravital imaging to investigate microvascular interactions of VlsE in gain of functions strains. **A)** Intravital imaging micrograph of *B*. *burgdorferi* in a post-capillary venule of mouse knee joint (see **Table 1** for strain info). An infectious GFP-expressing *B*. *burgdorferi* B31 strain (GCB726) was injected into the tail vein of BALB/c mice. Vascular adhesion interactions were detected by high acquisition rate spinning disk confocal intravital microscopy 5 min post-infection. Blood vessels were stained with Alexa Fluor 647 anti-mouse CD31 (PECAM-1) antibody, shown in red. **B)** The effect of VlsE (GCB3023) and VlsE_ECM_ (GCB3025) on transient microvascular interactions in a B31-A background (see **Table 1**). The control strain lacking VlsE was GCB3212. After infection, the number of interactions/minutes in transient adhesion interactions were analyzed using intravital microscopy (see Materials and Methods). Error bars represent SD and statistical significance was analyzed using the non-parametric two-stage linear step-up procedure of Benjamini, Krieger and Yekutieli (n = 4 mice). **C)** Clearance of *B*. *burgdorferi* from the vasculature. Clearance of *B*. *burgdorferi* from the vasculature in the mice used in panel B was monitored by withdrawal of blood through the tail vein at 3- and 18-minutes post-inoculation as described in [8] and spirochetes number in the plasma were counted by dark-field microscopy. The data are plotted as the percentage of spirochetes in plasma at 18 minutes relative to the 3 minutes time point for each mouse.

To investigate whether the DS binding activity of VlsE might play a role in murine microvascular interactions, we used an intravital microscopy (IVM) assay. High passage non-infectious and non-adherent *B*. *burgdorferi* B3-1A [32] was transformed with a GFP-expressing plasmid constitutively producing either wild-type VlsE or the VlsE_ECM_ mutant from the *flaB* promoter on a shuttle vector encoding GFP [10] (see **Table 1**). These strains were inoculated into mice through iv injection and within the first hour post-inoculation we examined transient interactions (tethering and dragging interactions, which required less than 20 seconds to travel 100 μM along the vessel wall) by IVM (**Fig 4A and S1 Video).** We found that VlsE but not VlsE_ECM_ promoted transient endothelial interactions in knee joint-proximal tissue, compared with the parental strain lacking VlsE (**Fig 4B)**. Finally, because the clearance rate of *B*. *burgdorferi* can affect the number of vascular interactions, we compared the spirochete clearance rate in the three strains. As shown in **Fig 4C**, the presence or absence of VlsE or VlsE_ECM_ did not change the clearance rate. The data from this figure indicate that VlsE has the capacity to promote transient interactions of *B*. *burgdorferi* with the murine microvasculature. The lysine residues changed in the VlsE_ECM_ mutant, which are involved in DS binding, are important for the microvascular interactions.

### VlsE is an adhesin that promotes transient interaction of *B*. *burgdorferi* with the microvasculature in a low-passage infectious strain

**Fig 5:**
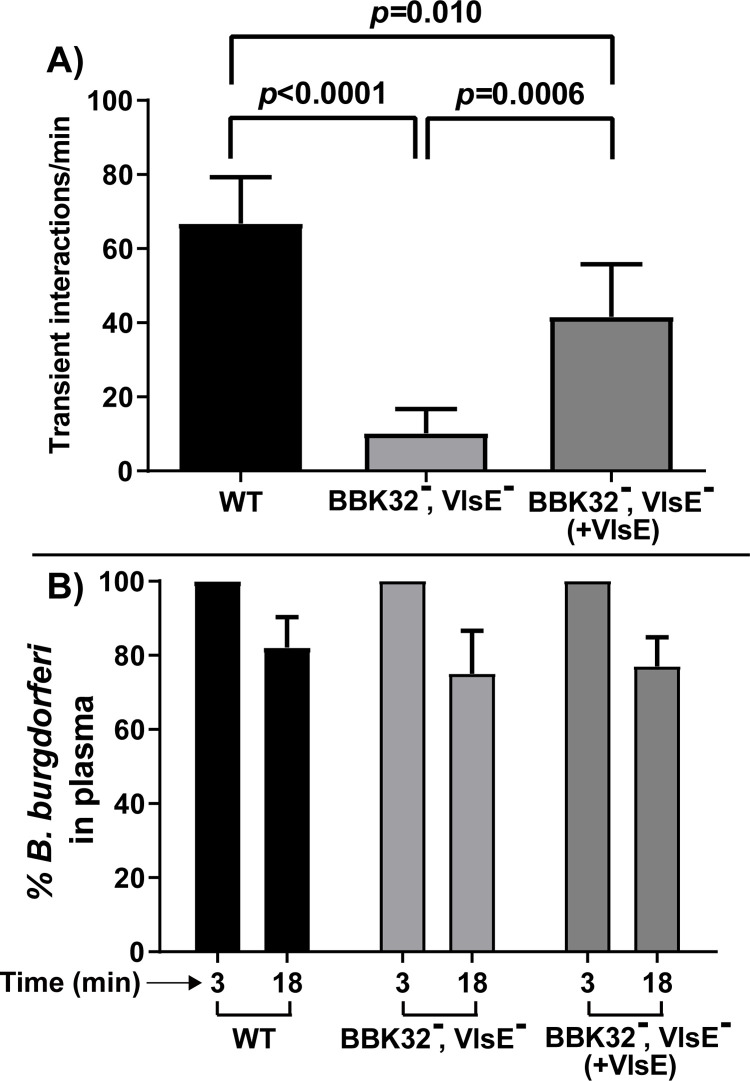
Intravital imaging to investigate microvascular interactions of a low-passage doubly deficient strain lacking VlsE and BBK32, and its VlsE complemented strain. Microvascular interaction rates in knee joint-proximal vessels are shown for three GFP-expressing *B*. *burgdorferi* strains, as analyzed by high acquisition rate spinning disk confocal intravital microscopy (see **Table 1** for strain info). **A)** The effect of wild type strain (GCB726), VlsE, BBK32 doubly deficient strain (GCB4036) and the doubly deficient strain complemented with VlsE (GCB4517) on transient microvascular interactions. Error bars represent SD and statistical significance was analyzed using the non-parametric two-stage linear step-up procedure of Benjamini, Krieger and Yekutieli (n = 4 mice). **B)** Clearance of *B*. *burgdorferi* from the blood in the infected mice used in panel A was monitored with the same method as in Fig 4C.

We next sought to understand if VlsE promoted vascular adherence in a low-passage infectious *B*. *burgdorferi* strain. This analysis is complicated by the presence of BBK32, a strong adhesin that promotes this process [11,12]. Disruption of the *bbk32* gene results in about a 50% reduction in transient interactions. Thus, to study the adhesin properties of VlsE in a low-passage infectious strain, we generated a doubly deficient strain lacking both *bbk32* and *vlsE* (GCB4036) by knocking out *bbk32* in a strain lacking lp28-1, which carries *vlsE* (**Table 1**). Using our IVM vascular interaction assay we found that the combined genetic removal of *bbk32* and *vlsE* reduced transient vascular interactions in the joint of mice by ~85% compared with the wild-type control strain (**Fig 5A** and **S2 Video)**. Complementation with a constitutively expressed *vlsE* gene restored transient interactions to 62.3% of the wild-type level, as expected for a strain lacking BBK32 [11]. No significant differences were found in the clearance rate (**Fig 5B)**. Therefore, combined with the data in **Fig 4**, we conclude that VlsE acts as vascular adhesin mediating transient interactions under shear force *in vivo*.

### DS-binding activity mediates VlsE-promoted transient microvascular interactions of *B*. *burgdorferi*

**Fig 6:**
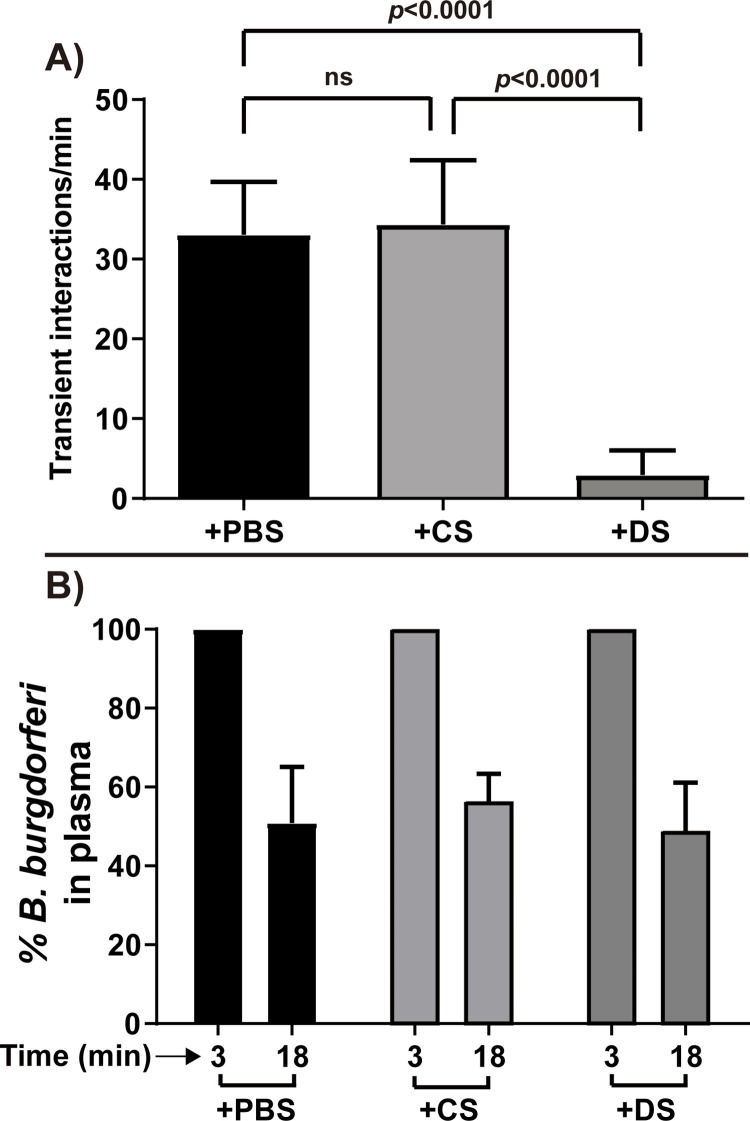
Intravital imaging to investigate the effect of DS on microvascular interactions of VlsE in a gain of function strain (see Table 1 for strain info). **A)** Mice were infected with a VlsE gain of function strain (GCB3023) and were injected either with 1.5 mg of DS or 1.5 mg chondroitin-4-sulfate (CS), or PBS into the tail vein of the *Cd1d*^*-/-*^ at the time of infection (n = 4 mice for each group). The number of interactions per minute for transient adhesions was analyzed using intravital microscopy (see Materials and Methods). Error bars represent SD and statistical significance was analyzed using the non-parametric two-stage linear step-up procedure of Benjamini, Krieger and Yekutieli. B) Clearance of *B*. *burgdorferi* from the vasculatures in the infected mice used in panel A was monitored with the same method as in Fig 4C.

The initiation steps of microvascular interactions *in vivo* involve FN and/or GAGs [10–12] and as shown in **Figs 1** and **2**, VlsE is able to bind to DS *in vitro*. Thus, to investigate the binding effect of DS on *B*. *burgdorferi* VlsE for vascular interactions, we infected mice with a GFP-labeled *vlsE* gain-of-function strain and at the time of infection i.v. injected DS or, as a negative control, chondroitin 4-sulfate which is structurally similar [38] but did not recognize VlsE *in vitro* (**Fig 1**). As expected, addition of DS but not chondroitin 4-sulfate significantly reduced the number of transient interactions (**Fig 6A**). No significant difference in the clearance rate was observed (**Fig 6B)**. Hence, the VlsE DS-binding activity contributes to *B*. *burgdorferi* transient interactions with the microvasculature.

### VlsE displays the properties of adhesin-X

**Fig 7:**
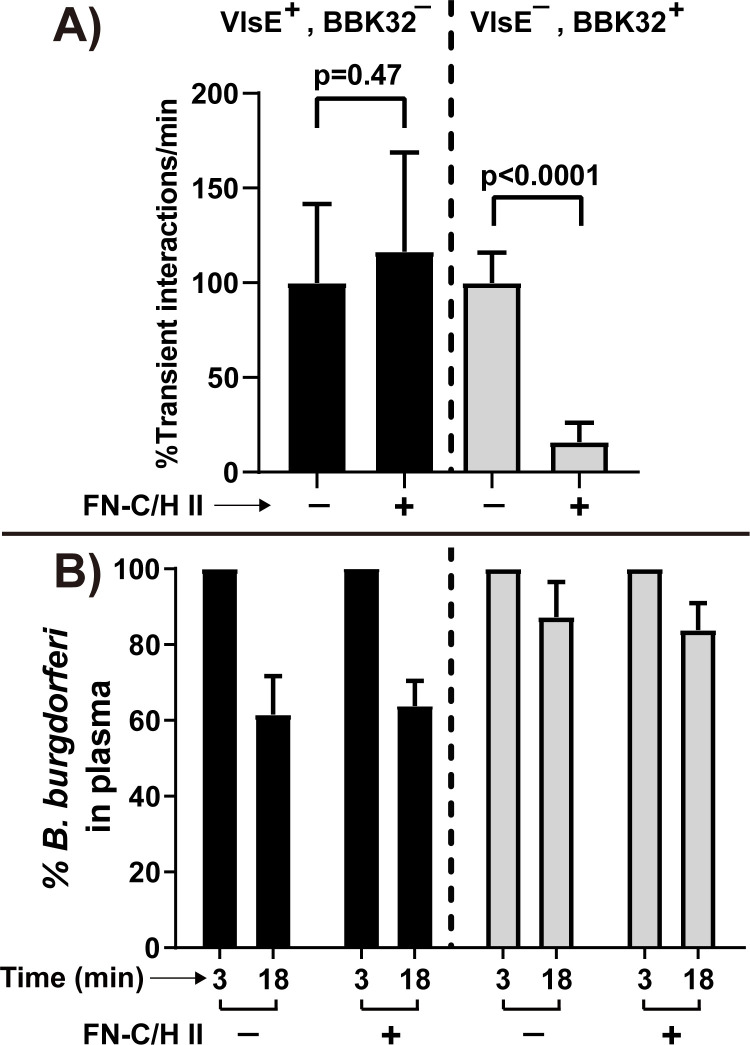
Intravital imaging to investigate the effect of the fibronectin heparin-binding peptide (FN-C/H II) on microvascular interactions in a VlsE gain of function strain (see Table 1 for strain info). **A)** Transient interaction rates in the knee joint were enumerated for two GFP-expressing B. *burgdorferi strains*: VlsE gain of function strain (GCB3023: VlsE^+^, BBK32^-^) and the control VlsE deficient strain (GCB4043: VlsE^-^, BBK32^+^) in mice treated or untreated with the FN heparin-binding peptide (FN-C/H II), as analyzed by high acquisition rate spinning disk confocal intravital microscopy. *B*. *burgdorferi* strains were prepared and injected as described in Materials and Methods, together with 100 μg of FN-C/H II peptide as [11]. The data are plotted as the percentage of transient interaction/min observed in mice treated with FN-C/H II relative to the untreated mice for each strain. Error bars represent SD and statistical significance was analyzed using the non-parametric Mann-Whitney test (n = 4 mice). **B)** Clearance of *B*. *burgdorferi* from the vasculatures in the infected mice used in panel A was monitored with the same method as in Fig 4C.

In our previous intravital studies [11], we reported that the GAG and FN-binding protein BBK32 is an important vascular adhesin in *B*. *burgdorferi*. However, disruption of the *bbk32* gene did not eliminate vascular interactions but reduced them by about 50%, leading to the hypothesis of the existence of at least one other efficient vascular adhesin, which we termed adhesin-X. Similarly, transient interactions of a low-passage BBK32-proficient *B*. *burgdorferi* strain could be inhibited by about 50% by injection of the FN C-terminal HepII heparin-binding peptide [11], consistent with the hypothesis that the BBK32 Fn-binding activity contributes to vascular adherence and is blocked by the FN HepII peptide [11]. In contrast, the residual vascular adherence of a BBK32-deficient *B*. *burgdorferi* strain that is mediated by adhesin-X was entirely unaffected by pre-injection of the FN HepII peptide. To determine if *vlsE* has this key property of adhesin-X, we analyzed the effect of the FN HepII peptide on *B*. *burgdorferi* vascular interactions in a *vlsE* gain-of-function strain, where BBK32 is absent (**Fig 7**). We found that addition of the FN HepII peptide did not reduce the number of transient interactions (**Fig 7A**). The effectiveness of the FN HepII peptide was demonstrated in the control knockout strain lacking VlsE but containing BBK32. Consistent with previous findings [11], in this strain an 85% decrease in transient interactions were observed in the presence of the FN HepII peptide. With or without FN HepII peptide treatment, there were no significant differences for the clearance rate (**Fig 7B)**. Therefore, VlsE appears to have the expected properties of adhesin-X and mediates a substantial portion (roughly 50% [11]) of the transient interactions observed upon iv inoculation of *B*. *burgdorferi*.

### A VlsE, BBK32 deficient strain is capable of vascular transmigration into peripheral joint tissue

**Fig 8:**
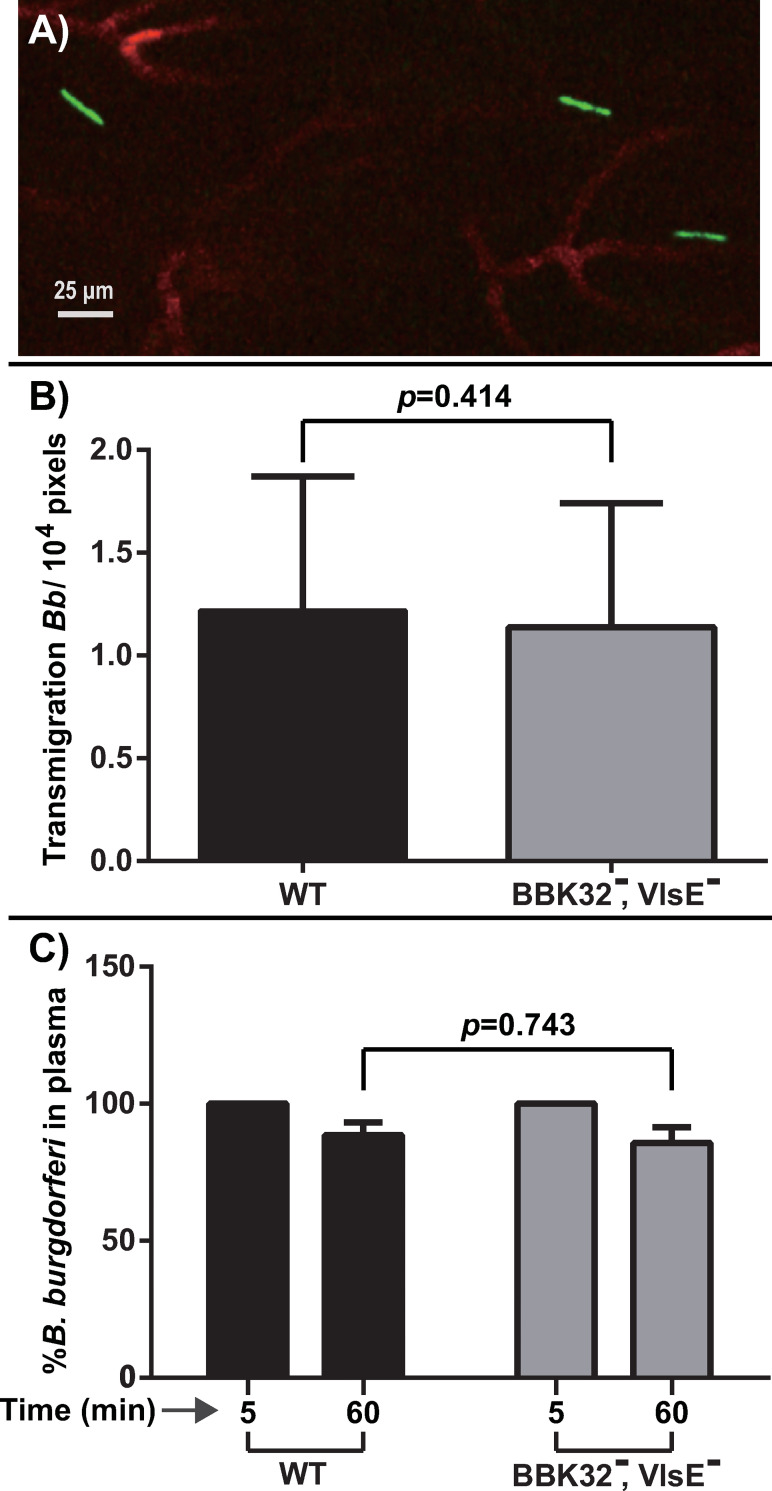
Intravital imaging to investigate the effect of a VlsE, BBK32 doubly deficient strain on vascular transmigration (see Table 1 for strain info). **A)** Intravital imaging micrograph of transmigrated spirochetes lacking both VlsE and BBK32. A GFP-producing doubly deficient strain (GCB4036) was injected into the tail vein of the *Cd1d*^*-/-*^ mice 24 hours before imaging. Blood vessels were stained with Alexa Fluor 647 anti-mouse CD31 (PECAM-1) antibody in red. B) Vascular transmigration was scored in the knee joint-proximal tissue by intravital microscopy of wild-type (GCB726) and the doubly deficient strain (GCB4036). The data were plotted as the number of transmigrated spirochetes observed in an area of 10^4^ pixels. Error bars represent SD and statistical significance was analyzed using the non-parametric Mann-Whitney test (n = 4 mice). C) Clearance of *B*. *burgdorferi* from the vasculature in the mice used in Panel B was monitored at 5 and 60 minutes after infection as described for Fig 4C.

Given the predominant roles of BBK32 and VlsE in initial vascular interactions, we tested a doubly deficient strain lacking both VlsE and BBK32 with dramatically reduced vascular interactions (~15% of WT) for its ability to undergo vascular transmigration into knee-joint peripheral tissue. We used our previously developed IVM vascular transmigration assay using *Cd1d*^-/-^ mice [8,14] where we observe extravasated spirochetes at 24 hours post-inoculation (**Fig 8A**). Interestingly, there was no significant difference in transmigration between the doubly deficient VlsE, BBK32 strain and the wild-type control (**Fig 8B**). Similarly, no significant difference in the clearance rate was observed between the two strains (**Fig 8C)**. Collectively, these observations indicate that decreased vascular interaction resulting from the absence of both BBK32 and VlsE have no apparent detrimental effect on *B*. *burgdorferi* extravasation from the knee joint microvasculature, and suggest that there might be another key adhesin involved in vascular transmigration of *B*. *burgdorferi*.

Consistent with the apparent lack of activity of either VlsE or BBK32 in extravasation, neither was required for tissue colonization in long-term mouse infection experiments. Mice were inoculated with strains GCB4080 (encodes *bbk32* and *vlsE*), GCB4036 (doubly deficient for *bbk32* and *vlsE*), GCB4517 (doubly deficient strain complemented with VlsE) and GCB4082 (doubly deficient strain complemented with VlsE_ECM_), then euthanized at 3 weeks post-inoculation. Tissue samples were collected distal to the inoculation site and the presence of viable spirochetes assessed by culture. Neither an obvious requirement for VlsE nor a deficiency from the VlsE_ECM_ mutation was observed (**Table 2**).

**Table 2:** Long-term mouse infections with wild-type and vlsE and bbk32 mutants. Infection of SCID mice inoculated with *B*. *burgdorferi* strains GCB4036, GCB4080, GCB4082, and GCB4517 (see Table 1) was assessed at three weeks post-inoculation by culture. Shown are the number of mice infected at each of two disseminated sites over the number of mice inoculated for each dose and B. burgdorferi strain.

		PBS		GCB 4080	GCB 4036	GCB 4517	GCB 4082
			Bbk32	+	-	-	-
dose delivered	tissue cultured		VlsE	+	-	+	VlsE_ECM_
	bladder	0/5					
	ear	0/5					
10^3^ bacteria	bladder			0/5	1/5	0/5	1/5
	ear			0/5	1/5	0/5	1/5
10^5^ bacteria	bladder			5/5	5/5	4/5	5/5
	ear			5/5	5/5	4/5	5/5
10^7^ bacteria	bladder			5/5	5/5	ND*	5/5
	ear			5/5	5/5	ND*	5/5

*Not determined. This set of mice was lost to a mechanical failure in the animal facility.

## Discussion

### VlsE is an adhesin with adhesive properties similar to both OspC and BBK32

The current work builds upon 1) a previous phage display screen identifying VlsE as a potential *B*. *burgdorferi* adhesin [23], 2) previous works dealing with the important *B*. *burgdorferi* adhesins BBK32 [25,39] and OspC [14,24], and 3) the involvement of protein-mediated microvascular interactions that promote hematogenous dissemination of *B*. *burgdorferi* [11,12,14,24,40,41]. This study shows that besides its well-established role in antigenic variation [16,17,22], VlsE is a DS binding adhesin. Notably, despite a lack of obvious sequence or structural similarities, VlsE displays properties similar to OspC and BBK32.

### OspC similarities

OspC is an important outer surface lipoprotein that binds to DS and FN [14]. It is required for extravasation into joint peripheral tissue in living mice and promotes joint colonization [14]. Identification of a lysine-rich DS binding region in OspC led us to look for a similar lysine cluster in VlsE and for a DS binding activity. We report here a DS binding activity for VlsE with an apparent K_D_ of 0.51 μM, similar to what we have reported for OspC (0.57 μM, see **Table 2**), although the kinetic binding parameters differ as discussed in a later section. Mutagenesis of the lysine residues in question in VlsE completely abrogated binding to DS as was previously observed for OspC. However, the OspC DS-binding activity does not appear to promote transient microvascular interactions as assessed by IVM (compare **Figs 4** and **S3**). Nonetheless, microvascular interactions must occur for OspC to be able to confer joint invasion and colonization [14]. In contrast, we found that the DS-binding activity of VlsE promotes transient vascular interactions, but previous findings shows that the *vlsE*-deficient strain retains full ability to colonize tissues in SCID mice [20,42]. One intriguing possibility is that the DS-binding activity shared by OspC and VlsE might allow one protein to mask the absence of the other protein under some conditions *in vivo*. This potential redundancy in function would be limited by the distinct onset-dependent production of these proteins: in immunocompetent mice, *ospC* expression begins after ticks feed and is downregulated shortly after *B*. *burgdorferi* disseminates, whereas the expression levels of *vlsE* are regulated by YebC [43] and are not significant until later, when spirochete dissemination occurs [44,45]. Nevertheless, in a SCID mouse, which lacks the adaptive immune response that is ordinarily countered by the hypervariable VlsE, persistent infectivity of a VlsE-deficient, but not a VlsE^+^ *B*. *burgdorferi* strain, requires the production of OspC [24], suggesting a partial redundancy in their functions. However, this notion does not rule out the possibility that the *in vitro* ligand-binding activities that are not shared by VlsE and OspC (e.g. the OspC C4b- or plasminogen-binding activities) confer non-redundant phenotypes *in vivo* [37,46,47].

### BBK32 similarities

BBK32 is a major FN and GAG binding adhesin [25,26] promoting transient interaction of *B*. *burgdorferi* to the microvasculature as shown by imaging in living mice or in flow chambers [11,12,27]. In a high-passage non-adherent *B*. *burgdorferi* strain BBK32 imparted efficient transient microvascular interactions. However, disruption of *bbk32* in an infectious background resulted in a loss of only roughly half of the transient interactions, leading us to suggest the existence of at least one other adhesin, referred to as adhesin-X [11]. In the experiments described here we present data supporting the identification of VlsE as adhesin-X. As does BBK32, VlsE imparts efficient transient microvascular interactions in a high-passage non-adherent *B*. *burgdorferi* strain **(Fig 4)**. These interactions are resistant to competition by the FN HepII peptide **(Fig 7)**, as previously described for adhesin-X; therefore, the observed VlsE microvascular interactions do not occur through FN. Finally, removal of both BBK32 and VlsE decreases transient microvascular interactions to background levels, indicating that the sum of the activity of these two adhesins constitutes the vast majority, if not the totality of detectable adhesin activities promoting transient microvascular interactions observable by intravital imaging **(Fig 5)**.

### Kinetic parameters of adhesin-DS interactions and the effect on resulting microvascular interactions

Although the primary ligands for BBK32 (FN) and VlsE (DS) differ, a possible hint as to their ability to promote microvascular interactions while other adhesins such as OspC do not, may come from their kinetic binding parameters (**Table 3).** Considering that *B*. *burgdorferi*-microvascular interactions must be established in the presence of the high forces of shear stress resulting from blood flow [10–12,27,48], adhesins with the ability to establish interactions most quickly would be more likely to do so with spirochetes trafficking through the microvasculature at a rate of greater than 100 μm/sec. As shown in **Table 3**, OspC and VlsE each bind to DS with similar affinity. OspC with a k_on_ of 1.71x10^4^s^-1^M^-1^ cannot promote transient microvascular interactions observable within the time-frame used for IVM (**S3 Fig**). In contrast, VlsE, with a very similar apparent K_D_, but a 35-fold higher association rate constant, very efficiently promotes transient interactions with the microvasculature, suggesting that the rate of association may be an important feature for establishing tethering interactions.

**Table 3:** Comparison of ligand binding parameters for OspC, VlsE and BBK32. The values noted are for proteins from *B*. *burgdorferi* B31 and are taken from the following sources: OspC, [14]; VlsE, this work; BBK32 (residues 45–68) and BBK32 (residues 108–205), [49]. The BBK32 proteins that were included for the kinetic parameters here are the fragments of the amino acids 45–68 and 108–205, which are the DS- and FN-binding domains of BBK32.

Protein	K_D_ (μM)	k_on_ (10^4^s^-1^M^-1^)	k_off_ (s^-1^)
OspC / DS	0.57 ± 0.17	1.71 ± 0.17	0.01 ± 0.004
VlsE / DS	0.51 ± 0.05	60.3 ± 7.5	0.31 ± 0.01
BBK32 (45–68) / DS	0.23 ± 0.039	14.5 ± 0.17	0.03 ± 0.17
BBK32 (108–205) / FN	0.018 ± 0.001	398 ± 0.15	0.072 ± 0.002

Notably, BBK32 promotes transient interactions in spite of a k_on_ for DS that is 4-fold lower than that for VlsE. However, the Fn-binding activity rather than the DS-binding activity of BBK32 promotes efficient microvascular interactions and displays an extremely high affinity (low K_D_) and high association rate constant. This activity is responsible for slowing the circulating spirochetes through tethering interactions, potentially removing the kinetic barrier and rapid association requirement for the BBK32-DS association [11,12,27]. These factors along with the involvement of soluble plasma FN [48] make a direct comparison of BBK32-Fn interactions with the DS binding properties noted in **Table 3**. Nevertheless, the extremely high K_on_ for BBK32-FN is consistent with our argument above that the association rate of an adhesin and its ligand is a crucial parameter governing the establishment of vascular interactions. Whether VlsE forms catch bonds, as does BBK32 remains to be investigated *in vitro* [27].

### Transient interactions and vascular transmigration

A perplexing question arising from our studies on BBK32 and VlsE is why a strain deficient for one or both of these adhesins is not deficient in vascular transmigration (**Fig 8**) and tissue colonization (**Table 2** and [41,49–51]). One possible explanation is that there is yet another unidentified/uncharacterized *B*. *burgdorferi* protein with overlapping function such as an adhesin that promotes the formation of very short-lived microvascular interactions that cannot be visualized within the exposure times required for IVM. OspC might fulfill this description as it does not display microvascular interactions (**S3 Fig**), yet it does play a role in both tissue tropism and extravasation [14]. Or an adhesin might exist that specifically interacts with activated or activated and potentiated endothelium apparent after 24 hours of *B*. *burgdorferi* infection [13]; such an adhesin would not have been observed in previous intravital experiments and may await detection and characterization. Further studies on DS binding adhesins in *B*. *burgdorferi* and their similarities and differences in function will provide fertile ground for the pathogenic properties of this important spirochete and should also be investigated in additional *B*. *burgdorferi* strains.

## Materials and methods

### Ethics statement and use of mice

Animal experimentation was carried out in accordance with the principles outlined in the most recent policies and *Guide to the Care and Use of Experimental Animals* by the Canadian Council on Animal Care. The animal protocols (AC16-0244 and AC20-0159) were approved by The Animal Care Committee of the University of Calgary. Wild-type BALB/c mice were purchased from Charles River (Wilmington, MA) and *Cd1d*^*-/-*^ mice in a BALB/c background (Jax #2962) were bred in-house at the Clara Christie Centre for Mouse Genomics at the University of Calgary. Mice of both genders between 6–8 weeks of age were used. Animal work performed at the Medical College of Wisconsin was performed under protocol AUA00002263 approved by the Institutional Animal Care and Use Committee and was compliant with the guidance of the Office of Laboratory Animal Welfare of the US National Institutes of Health. Fox Chase SCID mice (C.B-17 SCID) were purchased from Charles River Laboratories and were fed and watered ad libitum in the MCW ABSL2 facility.

### Antibodies and reagents

Alexa Fluor 647-conjugated anti-mouse CD31 (Clone 390) was from BioLegend Inc, San Diego, CA and the VlsE antibody (200-401-C33) was from Rockland Immunochemicals, Limerick, PA). The FN heparin-binding peptide was synthesized by Bio Basic Inc., Markham, ON, Canada.

### Bacterial strains and culture

The GFP-expressing *B*. *burgdorferi* strains and levels of antibiotics used for *Borrelia* culture are indicated used in this study are described in **Table 1**. Spirochete cultures in BSK-II medium containing 6% rabbit serum were inoculated from frozen glycerol stocks. The spirochete cultures were grown for 48 hours in 1% mouse blood at 35°C to a concentration around 5 x 10^7^/ ml in 50 ml BSK-II medium containing 6% rabbit serum as previously described [10]. The presence of blood in the cultures serves to host-adapt the spirochetes by inducing the synthesis of proteins that are upregulated in the tick or following infection [52]. Spirochetes in 50 ml were harvested by centrifugation at 6000 x g for 15 minutes at 4°C and washed twice with 100 ml of cold phosphate-buffered saline (PBS) as previously described [8] and resuspended to the density of 2 x 10^9^/ml spirochetes in cold PBS for mouse infection. The *Escherichia coli* strain strains were cultivated at 37°C in Luria-Bertani broth or agar (BD Bioscience, Franklin Lakes, NJ), with ampicillin (100μg/mL), or no antibiotics when appropriate.

### Plasmid constructions

#### Expression plasmid construction and generation of the VlsE_ECM_ mutant

To generate an expression plasmid encoding an N-terminal GST fusion of the VlsE B31 A3 protein, genomic DNA was prepared from *B*. *burgdorferi* strain B31 A3 [28]. The full length *vlsE* gene (lacking the putative lipoprotein signal sequence) was PCR amplified, with restriction sites added using Q5 Hot Start Master Mix (New England Biolabs) and the primers prMP131 and prMP132. Primer sequences are detailed in **S1 Table**. The PCR fragment was then inserted into the BamHI and SalI sites of pGEX4T2 expression vector (GE Healthcare Life Sciences) using T4 DNA Ligase (New England Biolabs). To generate the VlsE_ECM_ mutant, residues at positions 163, 167, and 170 of *B*. *burgdorferi* B31 A3 VlsE were first changed to methionine residues using overlap extension PCR and primers prMP138 and prMP139. Primers prMP144 and prMP145 were then used to generate the final K>M substitution at position 159. All ligation reactions were directly electroporated into *E*. *coli* DH5α. Carbenicillin resistant colonies were screened by BamHI and SalI restriction digest and gel electrophoresis on a 1% agarose gel run for one hour at 75 V. Clones containing the correct insert size were subjected to Sanger sequencing. The cloning of the pGEX-4T2 plasmid encoding *ospD* from *B*. *burgdorferi* strain B31 has been described previously [37]. Confirmed plasmids were subsequently used to transform *E*. *coli* BL21(DE3).

#### Plasmids for use in gain of function experiments in *B*. *burgdorferi*

The plasmids pTM61*strep*,*gfp*,*vlsE*_*A3*_ (prMP09) and pTM61*strep*,*gfp*,*vlsE*_*ECM*_ (prMP10) were constructed by inserting the wild-type B31 A3 [28] *vlsE* gene or the *vlsE*_*ECM*_ mutant gene (see above) into the AscI restriction site of pTM61*strep*,*gfp* [8] as follows: The 5’ end of the *vlsE* open reading frame (containing the putative lipoprotein signal sequence) was amplified by PCR using Q5 Hot Start Master Mix (New England Biolabs) and primers prMP141 and prMP135 (**S1 Table**). The primers were designed so that the generated DNA fragment would be used as the forward primer in a second PCR reaction, in conjunction with primer prMP140 to amplify full length *vlsE* alleles, using the pGEX4T2 expression vectors as a template. The full length *vlsE* alleles were inserted into the pTM61*strep*,*gfp* vector [8] in front of the *flaB* promoter using AscI restriction sites at both the 5’ and 3’ ends, followed by electroporation into *E*. *coli* DH5α. Streptomycin resistant colonies were screened by AscI restriction digest, and positive clones were analyzed for insert orientation by Sanger sequencing (Applied Biosciences). Confirmed plasmids were subsequently used to transform *B*. *burgdorferi* B31-A.

#### Plasmids for use in low-passage *B*. *burgdorferi* strains

**GCE3951:** pTM61 *kan*,*gfp* was constructed by digesting pTM61 *gent* (GCE1173) [10] with AvrII and MluI (New England Biolabs, Beverly, MA). The kanamycin resistance cassette was amplified from pBSV2 using the primers B2929 and B2930 with restriction sites added. Primer sequences are detailed in **S1 Table**. The PCR product was excised with AvrII and MluI and ligated into the pTM61 prepared as described above using T4 DNA ligase (New England Biolabs). The ligation mixes were directly electroporated into DH5α competent cells. Kanamycin resistant colonies were screened by AvrII and MluI restriction digest.

**GCE3823:** The knockout plasmid pMC117 (*Δbbk32*::*strep*) was constructed by amplification of the disrupted *bbk32* gene from GCB971 [8,53] using the primers B3030 and B3031 (**S1 Table)**. The PCR product was cloned directly in pJET (ThermoScientific, Waltham, MA) to generate GCE3823 (pMC117) (**Table 1).**

**GCE3828:** pTM61*kan*,*pncA* (pMC119-1), was constructed by digesting pTM61 *kan* (GCE3951) with AvrII (New England Biolabs, Beverly, MA). The *pncA* was amplified from B31 (GCB921) using the primers B3028 and B3032 with restriction sites added as AvrII and SpeI. Primer sequences are detailed in **S1 Table**. The PCR product was cloned directly in TOPO (ThermoScientific, Waltham, MA) as GCE3970 (pTX19), then excised with AvrII and SpeI and ligated into the pTM61*kan*,*pncA* (pMC119-1) prepared as described above. The ligation mixes were directly electroporated into DH5α competent cells. Clones containing the correct insert size were subjected to Sanger sequencing (University of Calgary).

**GCE3993:** pTM61*kan*,*pncA*,*vlsE*_*ECM*_ (pTX32) was constructed by digesting pTM61*kan*,*pncA*,*vlsE* (pMC156, GCE4004) with Xmal and AvrII. The *vlsE* gene was amplified from GCB3025 using the primers B3120 and B3121 with restriction sites added as Xmal and AvrII. Primer sequences are detailed in **S1 Table**. The PCR product was cloned directly in pJET (ThermoScientific, Waltham, MA) as GCE3994 (pTX33), then excised with Xmal and AvrII and ligated into pMC156 (GCE4004) to generate pTX32 (pTM61*kan*,*pncA*,*vlsE*_*ECM*_, GCE3993). The ligation mixes were directly electroporated into DH5α competent cells.

**GCE4004:** pTM61*kan*,*pncA*,*vlsE* (pMC156) was constructed by digesting pTM61*kan*,*pncA* (pMC119-1, GCE3828) with Xmal and AvrII. The *vlsE* gene was amplified from GCB3023 using the primers B3120 and B3121 with restriction sites added as Xmal and AvrII. Primer sequences are detailed in **S1 Table**. The PCR product was cloned directly in pJET (ThermoScientific, Waltham, MA) as GCE4001 (pMC154), then excised with Xmal and AvrII and ligated into pMC119-1 (GCE3828) to generate pMC156 (pTM61*kan*,*pncA*,*vlsE*, GCE4004). The ligation mixes were directly electroporated into DH5α competent cells.

Plasmid content for all low-passage strains constructed clone was assessed [54]. Plasmid content for non-infectious GCB909 (B31-A) derivatives, which are lacking many plasmids and were used in gain of function experiments were not assessed.

### Generation of recombinant proteins and antisera

The glutathione-S-transferase GST-tagged VlsE, VlsE_ECM_ or OspD in the *E*. *coli* strain BL21(DE3) were produced according to the manufacturer’s instructions (BD Bioscience, Franklin Lakes, NJ) [37]. The GST tag was then removed using thrombin protease (GE Healthcare) as described previously [55]. Antisera against OspD raised from OspD-immunized mice was obtained from Dr. Xin Li [55]. The antisera against VlsE were generated by immunizing four-week-old Swiss Webster mice with VlsE as described previously [56]. The ability of each of these antisera to recognize VlsE or OspD was verified using ELISA, as described [57].

### ECM-binding assay by qualitative ELISA

Qualitative ELISA for ECM binding by VlsE was performed as described [14]. In brief, one microgram of BSA (negative control; Sigma-Aldrich), human plasma FN (Sigma-Aldrich), mouse fibroblast laminin (Sigma-Aldrich), type I collagen from human skin (Sigma-Aldrich), type IV collagen from human placenta (Sigma-Aldrich), chondroitin-4-sulfate from bovine trachea (Sigma-Aldrich), DS from porcine intestinal mucosa (Sigma-Aldrich), or chondroitin-6-sulfate from shark cartilage (Sigma-Aldrich) was used to coat microtiter plate wells by incubating the plate overnight at 4°C. Then, 100 μl of 2 μM of untagged OspD (negative control) [37], or VlsE was added to the wells. Polyclonal sera against OspD [55] or VlsE at the dilution rate of 1: 200 were used as the primary antibodies, whereas HRP-conjugated goat anti-mouse IgG 1:1,000 (Seracare Life Sciences) was used as the secondary antibody to detect the ECM binding by each of these proteins. The plates were washed three times with PBST (0.05% Tween 20 in PBS), and 100 μl of tetramethyl benzidine (TMB) solution (ThermoFisher Scientific) was added to each well and incubated for 5 min. The reaction was stopped by adding 100μl of 0.5% hydrosulfuric acid to each well. Plates were read at 405nm using a Tecan Sunrise Microplate reader at 5min after the incubation (Tecan Life Science).

### Circular dichroism (CD) spectroscopy

CD analysis was performed on a Jasco 810 spectropolarimeter (Jasco Analytical Instrument, Easton, MD) under nitrogen. CD spectra were measured at room temperature (RT, 25°C) in a 1 mm path length quartz cell. Spectra of VlsE (10 μM) or VlsE_ECM_ (10 μM) were recorded in phosphate based saline buffer (PBS) at RT, and three far-UV CD spectra were recorded from 190 to 250 nm for far-UV CD in 1 nm increments. The background spectrum of PBS without proteins was subtracted from the protein spectra. CD spectra were initially analyzed by the software Spectra Manager Program (Jasco). Analysis of spectra to extrapolate secondary structures were performed using the K2D3 analysis programs [58].

### Surface Plasmon Resonance (SPR)

Interactions of VlsE or VlsE_ECM_ with DS were analyzed by a SPR technique using a Biacore T200 (Cytiva, Marlborough, MA). DS was biotinylated as previously described [56]. Ten micrograms of biotinylated DS were conjugated to an SA chip (Cytiva) as indicated previously [59]. A control flow cell was injected with PBS. For quantitative SPR experiments to determine DS-binding, ten microliters of increasing concentrations (0.97, 3.91, 15.63, 62.5, 250, 1000 nM) of VlsE or VlsE_ECM_ were injected into the control cell and the flow cell immobilized with DS at 10 μl/min, 25°C. To obtain the kinetic parameters of the interaction, sensogram data were fitted by means of BIAevaluation software version 3.0 (GE Healthcare), using the one step biomolecular association reaction model (1:1 Langmuir model), resulting in optimum mathematical fit with the lowest Chi-square values.

### Mouse infection for intravital microscopy

BALB/c or *Cd1d*^-/-^ mice were anesthetized by intraperitoneal injection of 200 mg/kg ketamine hydrochloride (Bimeda-MTC, Animal Health Inc., Cambridge, ON) and 10 mg/kg of xylazine hydrochloride (Bayer Inc., Toronto, ON). For vascular transmigration and adhesion assays, anesthetized *Cd1d*^-/-^ mice were secured in a mouse restrainer and inoculated by tail vein injection with 3 x 10^8^ spirochetes in 150 μl of phosphate buffered saline (PBS).

### Intravital imaging

#### Preparation of the mouse knee joint for intravital imaging

Mice were anaesthetized with a mixture of ketamine (200 mg/kg) and xylazine (10 mg/kg) through intraperitoneal injection. Preparation of the knee joint for intravital imaging was performed as previously described [13]. Briefly, to effectively imaging the knee, the hair and skin over the medial aspect of the knee joint was carefully removed by excision without causing bleeding. The exposed area was immersed in PBS to maintain moistness. A microscope cover slip was positioned on top of the exposed surgical area and secured by vacuum grease to the plastic knee-holder.

#### Vascular adhesion assay

The vascular adhesion interaction assay for *B*. *burgdorferi* by intravital microscopy was performed as previously described in [10–12]. Briefly, a GFP-expressing *B*. *burgdorferi* strain was injected into the tail vein of the *Cd1d*^-/-^ mice (3 x 10^8^ spirochetes per mouse). After bacterial injection, vascular adhesion interactions were examined in six to twelve unbranched post-capillary venules of 15–25 μm diameter in the knee and recorded within 18–45 min post-inoculation by intravital microscopy. Blood vessels were stained with Alexa Fluor 647 anti-mouse CD31 antibody in red. *Borrelia* strains were prepared and injected as described above, together with 100 μg of FN-C/H II peptide (Bio Basic Inc., Markham, ON, Canada), as previously described [11]. Intravital microscopy for adhesion interactions was performed in knee joint peripheral tissue as described above.

#### Vascular transmigration assay

The vascular transmigration assay for *B*. *burgdorferi* by intravital microscopy was performed as previously described [8,13,14]. Briefly, a GFP-expressing *B*. *burgdorferi* strain was injected into the tail vein of the *Cd1d*^-/-^ mice (3 x 10^8^ spirochetes per mouse). Twenty-four hours after bacterial infection, the total number of extravasated spirochetes in the knee joint-proximal tissue were enumerated in the whole surgical area of the knee joint. Blood vessels were stained with Alexa Fluor 647 anti-mouse CD31 antibody in red.

### Long-term mouse infection experiments

Mice: Five-week old female Fox Chase SCID mice (C.B-17 SCID) were obtained from Jackson Laboratories, Bar Harbor, ME and housed in the Medical College of Wisconsin BSL2 animal facility. The mice were fed and watered *ad libitum*. All procedures used were reviewed and approved by the Institutional Animal Care and Use Committee of the Medical College of Wisconsin.

#### Bacteria

Bacterial strains used are described in Table 1. Bacteria from frozen stocks were cultured at 33°C in Barbour-Stoenner-Kelly (BSK) II medium [60] with selective antibiotics as indicated. Antibiotics were used at concentrations of 50 μg/ml for streptomycin and 200 μg/ml for kanamycin. Upon reaching cell density of 1 X 10^7^–5 X 10^7^ cells/ml, analysis to confirm genomic plasmid presence was performed using multiplex PCR as previously described [54].

#### Inoculation of mice

Cultured organisms were washed in phosphate buffered saline (PBS) + 0.2% normal mouse serum (NMS), counted by darkfield microscopy, and diluted to 1 X 10^8^ cells/ml. C.B-17 SCID mice were inoculated subcutaneously between the scapulae with 1 X 10^7^, 10^5^, or 10^3^ bacteria delivered in 0.1 ml volume, or the vehicle control 0.1 ml PBS + NMS.

Three weeks following inoculation, mice were euthanized. Samples of mouse bladder and ear were collected and were placed in BSK medium supplemented with 50 μg/ml rifampicin, 20 μg/ml phosphomycin, and 2.5 μg/ml amphotericin B for culture-based analysis of infection. Cultures were incubated at 33°C and checked weekly for up to 8 weeks to assess presence of live Borrelia via darkfield microscopy. A mouse was considered infected when at least one culture was positive. Note that one cage of mice inoculated with 1 x 10^7^ *B*. *burgdorferi* strain GCB4517 was lost to an equipment failure in the animal facility.

## Supporting information

S1 Fig Quadruple amino acid mutations did not affect the structure of VlsE. Far-UV CD analysis of VlsE and VlsE_ECM_. The molar ellipticity, Φ, was measured from 190-250nm for 10μM of each protein in PBS buffer. The mean ± SD of the percentages of each secondary structure in VlsE and VlsE_ECM_ were calculated from the CD spectra obtained in three different experiments and shown at the top. No significant differences of the percentage of each secondary structure from VlsE and VlsE_ECM_ (P > 0.05, Mann–Whitney T test).
(PDF)

S2 Fig Expression and surface localization of VlsE, as determined by Western blotting. To assess expression and cell surface localization of VlsE in GCB3023 and GCB3025, spirochetes were incubated in the absence (−) or presence (+) of proteinase K (Prot. K). Following spirochete lysis, proteins were analyzed by SDS-PAGE and western blotting and probed with VlsE- and GFP-specific antibodies. GFP was used as a loading control.
(PDF)

S3 Fig The effect of the *ospC* WT and *ospC*_ECM_ mutant on vascular adhesion and clearance in a high- passage *B*. *burgdorferi* strain in BALB/c mice. Non-infectious GFP-expressing *B*. *burgdorferi* wild type (GCB706), the *ospC* wild type (GCB4458), and the *ospC*_*ECM*_ mutant strain (GCB4452) were injected into the jugular vein of BALB/c, 4x10^8^ spirochetes per mouse (n = 3 mice). Over a period of up to 60 minutes, microvascular transient interactions/min (tethering + dragging) **(A)** and stationary adhesions/min **(B)** were enumerated in the knee joint-proximal tissue by intravital microscopy using spinning disk laser confocal microscopy. Statistical significance was analyzed using the non-parametric Kruskal-Wallis test; ns denotes not significant. **(C)** Concentrations of *B*. *burgdorferi* in mouse plasma after iv injection. BALB/c mice were inoculated with *B*. *burgdorferi* through the jugular vein before imaging and blood was withdrawn at 3- and 18-minutes post-inoculation (n = 3 mice). Blood cells were allowed to settle overnight as described in Materials and Methods and spirochetes in the plasma were directly counted by dark-field microscopy. The change in spirochete concentration between 3 and 18 minutes was determined for each mouse as the percentage of spirochetes present at 18 minutes relative to the initial 3-minute time point. Statistical significance was analyzed using the non-parametric Kruskal-Wallis test; ns denotes not significant.
(PDF)

S1 Video VlsE promotes adhesion interactions in knee joint-proximal tissue of BALB/c mice. Experimental conditions are as described in the legend to **Fig 4**. The video was captured 5 to 45 min post-infection of each mouse by tail vein injection with 3x10^8^ GFP-expressing *B*. *burgdorferi*. The blood vessels were stained with Alexa Fluor 647 anti-mouse CD31 (PECAM-1) antibody, shown in red. Elapsed time is shown at the top right and the scale is at the bottom left.
(MP4)

S2 Video Loss of VlsE and BBK32 disrupt adhesion interactions on knee joint-proximal tissue of BALB/c mice. Experimental conditions are as described in the legend to **Fig 5**. Here, we used non-infectious strain (GCB705) as a negative control (see Table 1). The video was captured 5 to 45 min post-infection of each mouse by tail vein injection with 3x10^8^ GFP-expressing *B*. *burgdorferi*. The blood vessels were stained with Alexa Fluor 647 anti-mouse CD31 (PECAM-1) antibody, shown in red. Elapsed time is shown at the top right and the scale is at the bottom left.
(MP4)

S1 Table Primers used in this study. (PDF)

S1 Data GraphPad Prism files for data in this paper. (ZIP)
