# Supplementary material for: VlsE, the nexus for antigenic variation of the Lyme disease spirochete, also mediates early bacterial attachment to the host microvasculature under shear force
Source: PLoS Pathog. 2022 May 23;18(5):e1010511. doi: 10.1371/journal.ppat.1010511 (PMC9166660; doi:10.1371/journal.ppat.1010511)
Supplement: S1 Fig — Far-UV CD analysis of VlsE and VlsEECM. The molar ellipticity, Φ, was measured from 190-250nm for 10μM of each protein in PBS buffer. The mean ± SD of the percentages of each secondary structure in VlsE and VlsEECM were calculated from the CD spectra obtained in three different experiments and shown at the top. No significant differences of the percentage of each secondary structure from VlsE and VlsEECM (P > 0.05, Mann–Whitney T test). (PDF) [file ppat.1010511.s001.pdf]

	α-helix	β-strand	other
VlsE	60.7± 0.03%	0.9± 0.008%	38.2± 0.02%
$VlsE_{ECM}$	62.2± 0.01%	1.1± 0.003%	36.6± 0.01%

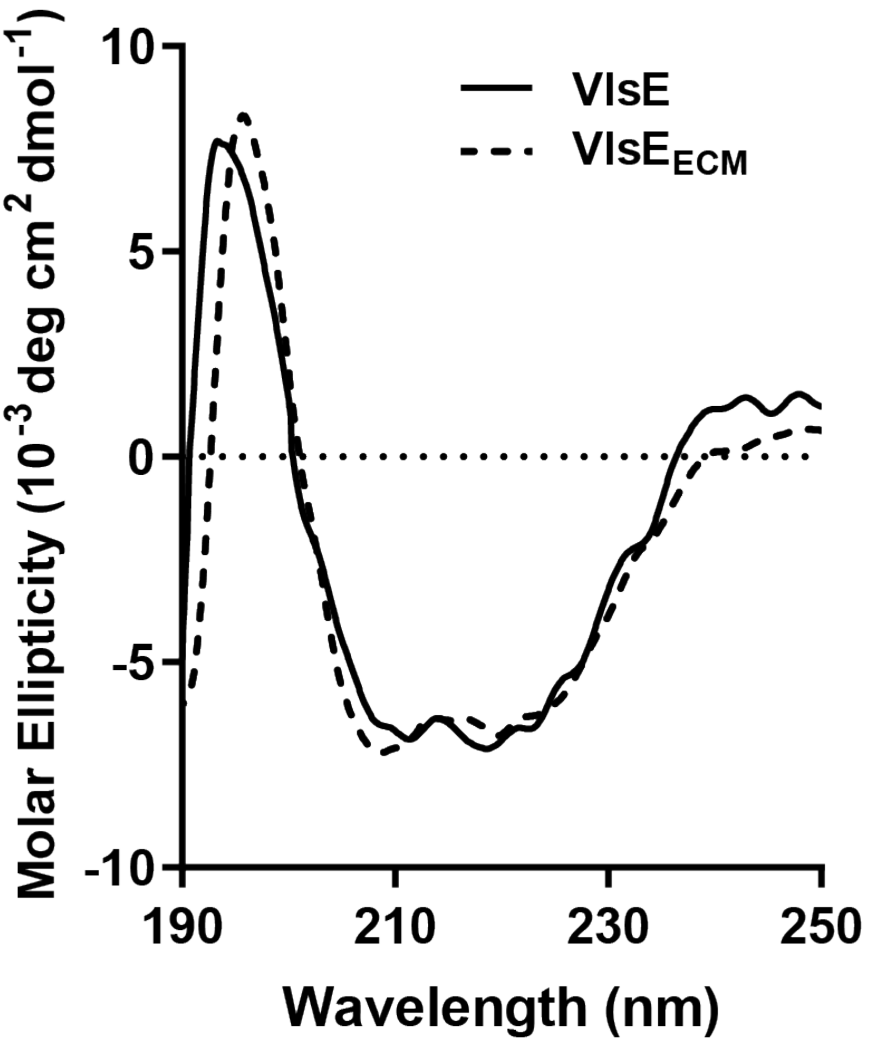

S1 Fig. Quadruple amino acid mutations did not affect the structure of VlsE. Far-UV CD analysis of VlsE and $VlsE_{ECM}$. The molar ellipticity, Φ, was measured from 190-250nm for 10μM of each protein in PBS buffer. The mean ± SD of the percentages of each secondary structure in VlsE and $VlsE_{ECM}$ were calculated from the CD spectra obtained in three different experiments and shown at the top. No significant differences of the percentage of each secondary structure from VlsE and $VlsE_{ECM}$ ($P > 0.05$, Mann–Whitney T test).
