# Supplementary material for: VlsE, the nexus for antigenic variation of the Lyme disease spirochete, also mediates early bacterial attachment to the host microvasculature under shear force
Source: PLoS Pathog. 2022 May 23;18(5):e1010511. doi: 10.1371/journal.ppat.1010511 (PMC9166660; doi:10.1371/journal.ppat.1010511)
Supplement: S2 Fig — To assess expression and cell surface localization of VlsE in GCB3023 and GCB3025, spirochetes were incubated in the absence (−) or presence (+) of proteinase K (Prot. K). Following spirochete lysis, proteins were analyzed by SDS-PAGE and western blotting and probed with VlsE- and GFP-specific antibodies. GFP was used as a loading control. (PDF) [file ppat.1010511.s002.pdf]

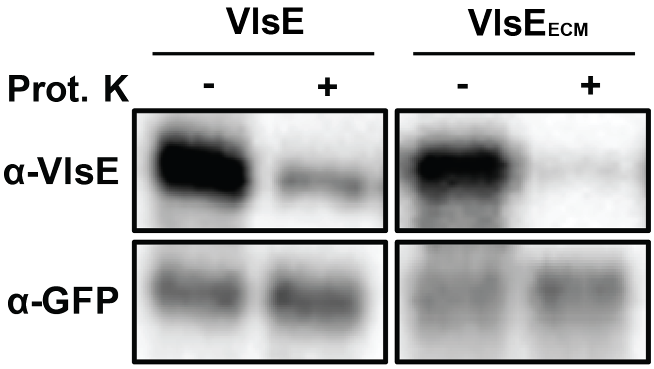

S2 Fig. Expression and surface localization of VlsE, as determined by Western blotting. To assess expression and cell surface localization of VlsE in GCB3023 and GCB3025, spirochetes were incubated in the absence (−) or presence (+) of proteinase K (Prot. K). Following spirochete lysis, proteins were analyzed by SDS-PAGE and western blotting and probed with VlsE- and GFP-specific antibodies. GFP was used as a loading control.
