# Supplementary material for: VlsE, the nexus for antigenic variation of the Lyme disease spirochete, also mediates early bacterial attachment to the host microvasculature under shear force
Source: PLoS Pathog. 2022 May 23;18(5):e1010511. doi: 10.1371/journal.ppat.1010511 (PMC9166660; doi:10.1371/journal.ppat.1010511)
Supplement: S3 Fig — Non-infectious GFP-expressing B. burgdorferi wild type (GCB706), the ospC wild type (GCB4458), and the ospCECM mutant strain (GCB4452) were injected into the jugular vein of BALB/c, 4x10^8^ spirochetes per mouse (n = 3 mice). Over a period of up to 60 minutes, microvascular transient interactions/min (tethering + dragging) (A) and stationary adhesions/min (B) were enumerated in the knee joint-proximal tissue by intravital microscopy using spinning disk laser confocal microscopy. Statistical significance was analyzed using the non-parametric Kruskal-Wallis test; ns denotes not significant. (C) Concentrations of B. burgdorferi in mouse plasma after iv injection. BALB/c mice were inoculated with B. burgdorferi through the jugular vein before imaging and blood was withdrawn at 3- and 18-minutes post-inoculation (n = 3 mice). Blood cells were allowed to settle overnight as described in Materials and Methods and spirochetes in the plasma were directly counted by dark-field microscopy. The change in spirochete concentration between 3 and 18 minutes was determined for each mouse as the percentage of spirochetes present at 18 minutes relative to the initial 3-minute time point. Statistical significance was analyzed using the non-parametric Kruskal-Wallis test; ns denotes not significant. (PDF) [file ppat.1010511.s003.pdf]

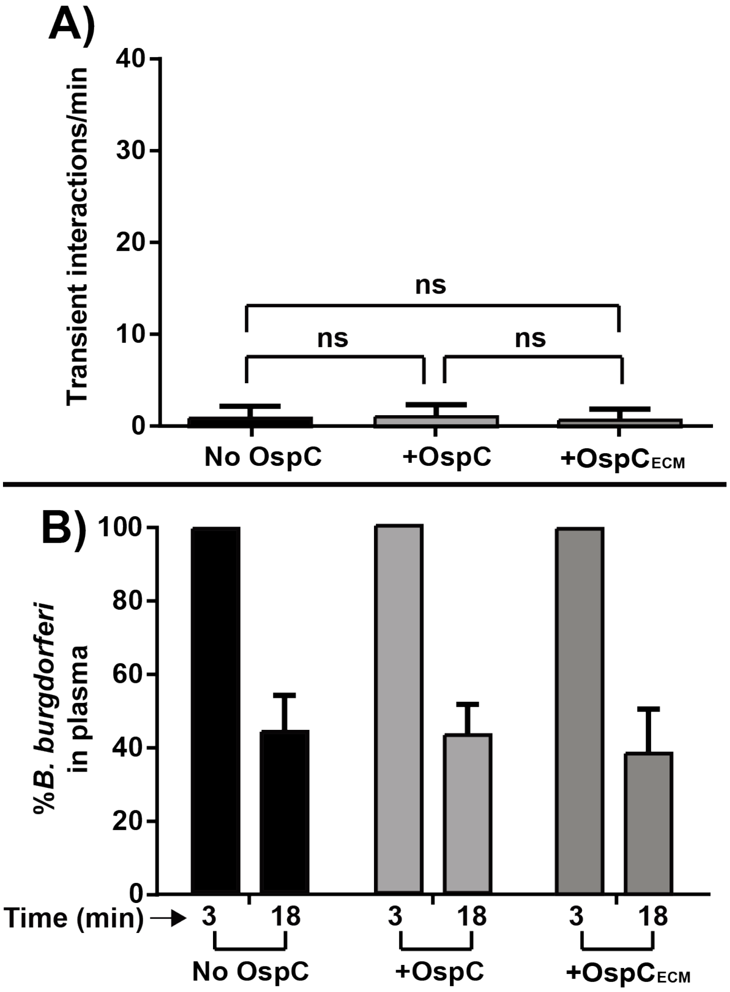

S3 Fig. The effect of the *ospC* WT and *ospC*$_{ECM}$ mutant on vascular adhesion and clearance in a high- passage *B. burgdorferi* strain in BALB/c mice. Non-infectious GFP-expressing *B. burgdorferi* wild type (GCB706), the *ospC* wild type (GCB4458), and the *ospC$_{ECM}$* mutant strain (GCB4452) were injected into the jugular vein of BALB/c, 4×10^8 spirochetes per mouse (n = 3 mice). Over a period of up to 60 minutes, microvascular transient interactions/min (tethering + dragging) **(A)** and stationary adhesions/min **(B)** were enumerated in the knee joint-proximal tissue by intravital microscopy using spinning disk laser confocal microscopy. Statistical significance was analyzed using the non-parametric Kruskal-Wallis test; ns denotes not significant. **(C)** Concentrations of *B. burgdorferi* in mouse plasma after iv injection. BALB/c mice were inoculated with *B. burgdorferi* through the jugular vein before imaging and blood was withdrawn at 3- and 18-minutes post-inoculation (n = 3 mice). Blood cells were allowed to settle overnight as described in Materials and Methods and spirochetes in the plasma were directly counted by dark-field microscopy. The change in spirochete concentration between 3 and 18 minutes was determined for each mouse as the percentage of spirochetes present at 18 minutes relative to the initial 3-minute time point. Statistical significance was analyzed using the non-parametric Kruskal-Wallis test; ns denotes not significant.
