# Supplementary material for: VlsE, the nexus for antigenic variation of the Lyme disease spirochete, also mediates early bacterial attachment to the host microvasculature under shear force
Source: PLoS Pathog. 2022 May 23;18(5):e1010511. doi: 10.1371/journal.ppat.1010511 (PMC9166660; doi:10.1371/journal.ppat.1010511)
Supplement: S1 Table — (PDF) [file ppat.1010511.s006.pdf]

S1 Table. Primers used in this study.

Primer name	Sequence (5'-3')
B2929	GCCCTAGGTACCCGAGCTTCAAGGAAG
B2930	GGACGCGTGTAAGCGCGCCGTCC
B3028	TTTCCTAGGTCTATGCTATCCCCTTGTTCA
B3032	TTTACTAGTTTTTTATATTGTGAGCCGGTTT
B3120	CCCCGGGTCACTTATTCAAGGCAGGAGGTGT
B3121	AGACCTAGG TGTCTGTCGCCTCTTGTGG
B3030	ATTACCCCCTGGTTTTA
B3031	AATATACCTTTTGTAAATTGACG
prMP131	GCTAGGGATCCGCTGATAAGGACGACC
prMP132	GCTAGGTCGACTCACTTATTCAAGGCAGG
prMP135	TGGTTTCCCCGTCGTACTACTTATATCGC
prMP138	CCCATAGCAATCCCCATCACACTCGCCATATCAGCAACCT
prMP139	GTGATGGGGATTGCTATGGGGATAATGGAGATTGTTGAAG
prMP140	GCTAGGGCGCGCCTCACTTATTCAAGGCAGGAGGTGT
prMP141	GCTAGGGCGCGCCCATTATAAGGAGACGATGAATATG
prMP144	GGCTGCTGATATGGATAGTGTGAT
prMP145	ATCACACTATCCATATCAGCAGCC

Restriction endonuclease sites are underlined.
